# Adaptation and validation of the Genetic Counseling Outcome Scale for autism spectrum disorders and related conditions

**DOI:** 10.1002/jgc4.1323

**Published:** 2020-09-06

**Authors:** Afiqah Yusuf, Iskra Peltekova, Tal Savion‐Lemieux, Jennifer Frei, Ridha Joober, Jennifer Howe, Stephen W. Scherer, Mayada Elsabbagh

**Affiliations:** ^1^ Azrieli Centre for Autism Research, Montreal Neurological Institute‐Hospital McGill University Montreal QC Canada; ^2^ Research‐Institute of the McGill University Health Centre Montreal QC Canada; ^3^ Department of Psychiatry McGill University Montreal QC Canada; ^4^ Douglas Mental Health University Institute Montreal QC Canada; ^5^ The Centre for Applied Genomics Hospital for Sick Children Toronto ON Canada; ^6^ McLaughlin Centre and Department of Molecular Genetics University of Toronto Toronto ON Canada

**Keywords:** autism spectrum disorder, empowerment, genetic testing, patient‐reported outcome measures

## Abstract

The genetics care pathway experienced by families affected by autism spectrum disorder (ASD) around the time of diagnosis is currently uncharacterized and potentially variable across contexts. The lack of consensus on outcome measures to capture the impact of genetic services for these families shows a gap in understanding and optimizing this genetics care pathway. The Genetic Counseling Outcome Scale (GCOS‐24) is a validated outcome measure of clinical genetics services. The current study aims to adapt and validate the GCOS‐24 as an outcome measure in the context routine genetic testing in ASD and related conditions. Families seen for their child’s developmental evaluation for ASD and related conditions were invited to participate in a genomics cohort between 2016 and 2018. Families (*n* = 111) completed the mGCOS‐24 (modified GCOS‐24), adapted from the original GCOS‐24 by clinicians working in the target population’s routine care pathway. The mGCOS‐24 has acceptable internal consistency (Cronbach’s α = 0.84) and high test–retest reliability (ICC = 0.88). It also inversely correlates with stress as measured by Perceived Stress Scale (PSS‐10) and distress, as measured by the Distress Thermometer, *r*s ≥ 0.39, *p*s < 0.001. The mGCOS‐24 had adequate readability, as supported by cognitive interviews completed by a sub‐sample of five mothers of a child with ASD. Together, our findings show that the mGCOS‐24 has good validity for the target population. Preliminary characterization of the genetics care pathway in this population revealed remarkable variability in pre‐test counseling and limited post‐test counseling. The use of the mGCOS‐24 as an outcome measure is useful in filling some of these gaps by offering a way to assess, and in the future, optimize the genetics care pathway for families affected by autism and related neurodevelopmental conditions.

## 1 INTRODUCTION

Autism spectrum disorders (ASD) is a life‐long neurobiological developmental disorder characterized by impairments in social skills and communication, narrow and intense interests, and repetitive behavior (American Psychiatric Association, 2013). Global prevalence estimates suggest that 1% to 2.6% of children have ASD (Elsabbagh et al., 2012; Kim et al., 2011). The essential steps of care involved in diagnosing ASD, *that is,* the diagnostic *care pathway* for ASD, extends across specialties and settings and is complex due to the heterogeneity of ASD clinical presentations and needs (Anagnostou et al., 2014; Johnson & Myers, 2007). This diagnostic care pathway requires a detailed developmental history from parents, teachers, and/or other caregivers, evaluation of the core symptoms of ASD, a thorough appraisal of abilities, and biological testing to rule out or monitor co‐morbidities, all of which are crucial not only for a diagnosis, but also to tailor medical and behavioral provisions the child will receive in clinic, in early intervention programs and/or in schools (Anagnostou et al., 2014; Johnson & Myers, 2007). This time‐intensive care pathway is experienced as a ‘diagnostic odyssey’ to individuals with ASD and their families due to uncoordinated and delayed access to the needed multiple specialties and settings (Lappé et al., 2018). This not only leaves individuals and families distressed, confused, and frustrated, but hinders immediate access to further care (Lappé et al., 2018) potentially exacerbating the autistic person’s developmental trajectory (Elsabbagh, 2020).

To accelerate the autism diagnostic care pathway, progress in ASD genetics research (Devlin & Scherer, 2012) has led to recommendations for offering chromosomal microarray (CMA) to families following a diagnosis of ASD as a ‘first‐tier’ test to provide an etiological explanation for the condition (Miller et al., 2010; Waggoner et al., 2018). Subsequently, exome sequencing has been recommended as an even more powerful genetic test, relative to CMA (Srivastava et al., 2019), and will soon replace the CMA in routine services. These genetic tests can accelerate the provision of care for individuals with ASD by (a) ruling out, allowing for monitoring of, and treating co‐morbidities to ASD and (b) providing an etiological explanation for the condition; thus, ending the diagnostic odyssey for these individuals and their families.

Despite the large proportion of CMA testing being done on this prevalent condition, the *genetics* care pathway in ASD for which genetic testing is embedded remains elusive, with a lack of consensus across different guidelines targeted to different specialties on varying details of care. Taken together, all guidelines recommend at least a medical assessment that includes the provision of genetic testing and some form of genetic counseling following certain types of genetics results (Miller et al., 2010; Schaefer & Mendelsohn, 2013; Volkmar et al., 2014). The most comprehensive recommendation (Schaefer & Mendelsohn, 2013) suggests the following care pathway: (a) referral by the primary care provider for CMA coupled with a tailored discussion of the family’s expectations for possible results of testing for every person with ASD, (b) genetic counseling to disclose and discuss the genetics results regardless of whether or not the CMA led to an identified genetic etiology for the individual, (c) depending on the specific result, referral to medical genetics, and lastly (d) further management of care as needed coordinated by both the geneticist and the primary care provider.

The extent to which this care pathway has been implemented is questionable. This is evidenced by the remarkably diverse genetics care pathways that both families have reported to experience and that care providers have reported to practice (Barton et al., 2018). No two families experienced the same care pathway to receive genetic testing; for example, some families were seen by different combinations of physicians prior to being offered varying combinations of genetic tests by different professionals, if offered at all (Barton et al., 2018). The level of care received at different steps of the care pathway is also discouraging. The majority of parents of children with ASD did not receive needed information about genetic testing during the referral for CMA by the primary care provider (Zhao et al., 2019) and may not undergo any genetic counseling prior to testing (Reiff et al., 2013). Access to medical genetics following genetics results is variable; the majority of families did not receive counseling following genetic results (Peabody et al., 2015), and more than 70% of caregivers of children with ASD surveyed in the USA and Mexico reported to never having seen a genetics professional at all despite having interest in testing (Codina‐Sola, Perez‐Jurado, Cusco, & Serra‐Juhe, 2017; Wydeven, Kwan, Hardan, & Bernstein, 2012). Taken together, it is clear that the genetics care pathway for ASD is in practice suboptimal.

Further, consistent and tailored genetics care for families across the care pathway is vital considering that the *clinical utility,* that is, the balance of benefits *vs*. risks of genetic testing, is a matter of ongoing debate. Clinical utility has thus far been measured using diagnostic yields and clinical outcomes. The diagnostic yields of CMA and exome/genome sequencing are relatively high (20% and ~40%‐68%, respectively (Clark et al., 2018; Farnaes et al., 2018; Malinowski et al., 2020; Scocchia et al., 2019)). The impact of clinical management is promising: CMA has estimated to alter treatment plans in 27%–54% of cases (Clark et al., 2018; Malinowski et al., 2020; Scocchia et al., 2019; Stark et al., 2019)) and to prevent infant morbidity in 61% of acutely ill infants (Farnaes et al., 2018). In sum, the clinical utility, if based solely on these diagnostic yields and clinical outcomes, suggests that integrating CMA and exome/genome sequencing in the care pathway for this population is overall positive.

However, we have argued that a ‘focus on health outcomes is inadequate in understanding overall net benefits versus risks’ (Yusuf et al., 2020), considering the range of *personal utility* of genetic testing, that is, psychosocial outcomes of genetic testing (Foster, Mulvihill, & Sharp, 2009), which range from coping, value of information, the ability for future planning, research altruism, etc., as identified across multiple conditions (Kohler, Turbitt, & Biesecker, 2017). In contrast, evidence for such impacts in neurodevelopmental conditions in general is more sparse. A recent systematic review found limited studies assessing psychosocial impact following exome/genome sequencing in individuals and families affected by developmental delay/intellectual disability or congenital anomalies (Malinowski et al., 2020). Consistent findings from mostly qualitative studies suggest that genetic results may offer clear benefits to affected families, such as relief, access to services, family planning, hope, revised care plans, etiological explanation, and increased understanding (Giarelli & Reiff, 2015; Hayeems, Babul‐Hirji, Hoang, Weksberg, & Shuman, 2016; Kiedrowski, Owens, Yashar, & Schuette, 2016; Reiff et al., 2012; Reiff et al., 2015; Wynn et al., 2018). On the other hand, several actual or perceived risks of genetic results in neurodevelopmental conditions have also reliably been identified. For example, negative or variants of uncertain significance (VUS) results were perceived as disappointing in not providing answers to their child’s condition (Giarelli & Reiff, 2015) and even adding more uncertainty about the condition’s etiology (Hayeems et al., 2016; Reiff et al., 2012; Reiff et al., 2015). Further, higher distress levels and uncertainty have been reported by parents who interpreted their child’s results as positive compared with those who interpreted them as negative following clinical exome sequencing (Wynn et al., 2018); in addition, some parents also expressed guilt because they may have passed on the variant to their child (Kiedrowski et al., 2016; Reiff et al., 2015). Other negative emotions have also been reported by parents, namely: shock, devastation, fear, and sadness (Kiedrowski et al., 2016). Taken together, it is possible that the personal utility of genetic testing varies by the type of genetic result received. It is also possible that the genetics care pathway can affect personal utility of genetic testing. A meta‐analysis of studies in which genetics results were returned in‐person to participants of different conditions reported overall low levels of distress and ‘positive emotional impact’ in pediatric patients in particular (Robinson et al., 2019). While some findings were consistent across multiple qualitative studies, suggesting the reliability of the findings, a lack of systematic methodology employing quantitative measures continues as a knowledge gap in describing and/or measuring outcomes of the genetics care pathway in ASD and related conditions toward optimizing personal utility of genetic testing in this population.

One way to address these gaps is to build on the progress made in the broader area of research in which patient‐reported outcome measures have been developed, deployed, and validated for clinical populations attending a *clinical genetics service*. A clinical genetics service is conceptualized as a counseling process involving at least two in‐person visits with a genetics professional for individuals deemed eligible for genetic testing—an initial visit to discuss the need for genetic testing (also known as pre‐test counseling) and a post‐test visit to disclose test results and their implications for the individual and family. Building on qualitative theory, empowerment has been proposed as a multi‐dimensional outcome from using clinical genetics services, including genetic counseling with or without genetic testing (McAllister, Dunn, & Todd, 2011; McAllister et al., 2008). McAllister *et al*. defined the construct of empowerment as the belief that an individual has derived benefits from their use of a clinical genetics service in terms of decisional, cognitive, and behavioral control, emotional regulation, and hope (McAllister, Dunn, et al., 2011; McAllister et al., 2008; McAllister, Wood, Dunn, Shiloh, & Todd, 2011).

The Genetic Counseling Outcome Scale (GCOS‐24) (McAllister, Wood, et al., 2011) has been validated in recent years as a patient‐reported outcome measure of empowerment. The GCOS‐24 was validated in a total of 395 patients prior to and following their first visit to a genetics clinic (*i.e.,* pre‐test counseling) where they were seen by genetics professionals (McAllister, Wood, et al., 2011). The measure showed good internal consistency (Cronbach’s α = 0.87), test–retest reliability (*r* = 0.86), and sensitivity to change over time (Cohen's *d* = 0.70) (McAllister, Wood, et al., 2011). There is strong evidence for construct validity: GCOS‐24 scores increased after attending pre‐test counseling were significantly higher in patients active in a support group and were significantly correlated with validated measures of health locus of control, perceived personal control, anxiety, depression, satisfaction with life, and authenticity (McAllister, Wood, et al., 2011; Thomas & McAllister, 2019).

Moreover, there is growing evidence for the validity and reliability of the GCOS‐24 as an outcome measure to capture the impact of genetics services for a wide range of clinically referred populations. Despite its advantages in addressing current gaps in research on ASD and related conditions, two reasons limit the usability of the GCOS‐24 within this population. First, the GCOS‐24 was developed for patients receiving genetic services themselves rather than for a family context, such as with ASD, where the genetics care pathway involves the affected child along with their caregiver and other family members. Second, the GCOS‐24 has been deployed in care pathways specific to *clinical genetics services* (Davison et al., 2017; Diness et al., 2017; Muñoz‐Cabello et al., 2018), rather than the context of first‐tier genetics testing in ASD. The former typically involves pre‐test and post‐test counseling all provided by a medical geneticist or genetic counselor targeting patient populations for whom a genetic condition is already suspected (Cohen et al., 2012). In contrast, the latter pathway involves a more diverse group of physicians (*e.g.,* family doctors, developmental pediatricians, and psychiatrists) ordering the test to primarily parents whose child is undergoing a diagnostic evaluation for a neurodevelopmental condition. Disclosure of genetics results to families and subsequent referral pathways based on the above literature is also less consistent.

Therefore, the current study addresses two aims: the primary aim was to adapt and validate the GCOS‐24 for caregivers of a children with ASD and related conditions using a large and representative cohort of healthcare users in a tertiary hospital setting. We assessed internal consistency, test–retest reliability, and construct validity of the adapted measure using a similar approach to the original GCOS‐24 (McAllister, Wood, et al., 2011). The secondary aim was to examine characteristics of the care pathway in which genetic testing is embedded and how it compares to existing recommendations.

## 2 MATERIALS AND METHODS

The multi‐site study was approved by the Research Ethics Board (REB) of the McGill University Health Centre and the REB of the Douglas Mental Health University Institute. Informed consent for participating in the study was obtained from all individuals, and the abovementioned REBs authorized the use of minimal de‐identified aggregate data of referred individuals who declined participation in the study.

### 2.1 Participants

Families were recruited into the *Genome to Outcome* (G2O) Cohort that aimed to examine child, family, and health service moderators of the impact of genetic results on parents of a child with a neurodevelopmental condition. It also integrated participation in a large‐scale genomics research study whose purpose was exploring the genetics of individuals with ASD and related conditions and was independent from this current study (Yuen et al., 2017; Jiang et al., 2013; Yuen et al., 2016; Yuen et al., 2015). Families eligible for the G2O Cohort were those with children between the ages of 0 to 18 diagnosed with a neurodevelopmental condition for which CMA was recommended. Children with a previously diagnosed genetic condition were excluded.

### 2.2 Procedures

Referrals to the G2O Cohort were made by 11 clinicians across several departments (Child Development, Genetics, and Psychiatry) in two hospitals: the McGill University Health Center and Douglas Mental Health University Institute. All participating clinicians were involved in a genetics care pathway for ASD either through a comprehensive diagnostic evaluation that includes CMA or a referral for CMA that followed a diagnosis of ASD from another clinician. Due to the CMA recommendation being inclusive of ASD‐related conditions like developmental delay/intellectual disability (DD/ID) (Miller et al., 2010), along with the similar care pathway that this population undergo, this study was inclusive of families who have a child with an ASD‐related conditions such as DD/ID.

Previous research has shown that clinician involvement in research studies as referees of potential participants and as co‐designers in the protocol to adapt the research protocol to best fit with their clinical practice, resulted in improved recruitment rate of the research sample, an approach we termed the ‘clinically integrated protocol’ (Bell‐Syer, Thorpe, Thomas, & Macpherson, 2011). Therefore, we aimed to achieve a representative sample from this approach in order to address the tendency for research sample participants to be of higher socioeconomic status and functioning compared to the general population (Mottron, 2004; L. Robinson, Adair, Coffey, Harris, & Burnside, 2016). We implemented these elements as a clinically integrated protocol, embedded within the care pathway experienced by families following a diagnosis of ASD or related conditions. This clinically integrated protocol consists of the following elements: (a) referring clinicians was actively involved in the design and implementation of the protocol, (b) a subgroup of the referring clinicians acted as ‘champions’ to support the integration of research within their service, and finally (c) referring clinicians introduced the research project to a potential participant during a clinical appointment to discuss the need for a clinical CMA during a disclosure of the child’s diagnosis.

Individuals interested in participating in the G2O Cohort agreed to be contacted independently by the research team within 1 week. Interested families were invited to a study visit at the hospital site, where they were initially recruited, and informed consent was obtained. As part of the consent procedure, participants agreed to grant the research team access to their child’s medical charts to complete the diagnostic information and characterize the genetics care pathway related to the CMA provision.

To reduce the burden on the family, another member of the research team was present to support the child and his/her sibling(s) in the same room while the caregiver(s) were consented. The caregiver ‘most knowledgeable’ about the child was asked to complete the online self‐report measures, detailed below in study measures. All data measures were available in English and French. Family members who consented to participate in the study then underwent genomics testing for research purposes. Another important element of the clinically integrated protocol is that the blood drawn for the clinically ordered CMA was performed on the diagnosed child concurrently with the draw for the genetic research sample. All measures in this study were collected following a referral for a CMA from the family’s clinician and prior to receiving genetic results.

Following the study visit, a subset of the participants who were considered highly engaged in research were invited to participate in cognitive interviews (Drennan, 2003) to verify that the modified GCOS‐24 was interpreted consistently across participants A minimum sample size of five for cognitive interviews is considered acceptable, provided that the potential for bias is acknowledged and the participants should consist of the target population who would complete the questionnaire (Willis & Artino, 2013). The cognitive interviews were conducted in‐person, and participants were asked to verbalize their thought process while reading each of the questionnaire’s 24 items, with the help of verbal probing from the interviewer. The interview was audio‐recorded and transcribed offline. During the interview, probes were used to ensure that all participants were asked the same question consistently. Probes for each item are as follows: (a) *Can you repeat the statement in your own words?* (b) *How did you arrive at that answer?* and (c) *Was this hard or easy to answer?*

### 2.3 Study measures

*Parent empowerment* was measured using the modified version of the GCOS‐24 (McAllister, Wood, et al., 2011) (mGCOS; Online Resource). A developmental pediatrician (IP) and a psychologist (TSL), both specializing in ASD and in the ASD care pathways, reviewed the original GCOS‐24, identified items requiring revision, and revised the items. Table 1 lists the four items of the original GCOS‐24 that required revision to adapt it for our target context as well as the justification for revising each item. We followed established guidelines to translate the mGCOS‐24 into French for the target population (Wild et al., 2005). This consists of (a) forward translation from English to French by a bilingual health professional familiar with the target population, (b) back‐translation from the translated French version into English by another bilingual health professional who is blind to the original English version, (c) reconciliation of the original English version, the translated French version, and the back‐translated English version of the questionnaires by the two translators for ‘semantic, idiomatic, experiential and conceptual equivalence’ (Diness et al., 2017) especially for items identified in the back‐translation to be problematic or ambiguous, and lastly (d) a final proof‐reading of the French version by another bilingual health professional. These guidelines are well‐established, especially in ASD research, and do not require labor‐intensive stand‐alone validation of the translated version, which has arguably hindered progress in autism research and care internationally (Durkin et al., 2015; Elsabbagh et al., 2014).

**TABLE 1:** Specific items from the Genetic Counseling Outcome Scale (GCOS‐24) that were revised for the modified Genetic Counseling Outcome Scale (mGCOS‐24)

Original	Revised	Justification(s)
I am clear in my own mind why I am attending the clinical genetics service	I am clear in my own mind why my family is having genetic testing	The referral to undergo genetic testing by the non‐genetic clinician is part of the disclosure of the child’s diagnosis and thus may not be perceived as a distinct ‘clinical genetics service’ to the family The majority of the target population do not get referred to geneticists/genetic counselors after undergoing genetic testing. We believe that simplifying the items to refer to one component of the service ubiquitous to all families in this population (i.e., undergoing genetic testing) was needed Finally, we initially considered ensuring that the concept of ‘clinical genetics service’ is consistently understood to all families to encompass the referral to undergo genetic testing. However, considering points (a) and (b), this requires differentiating certain aspects of the service the families have experienced over others even when many other components occur within one clinical encounter. Specifically, ‘clinical genetics service’ begins when the clinician suggests the family to undergo genetic testing for their child and ends following genetic counseling after genetic results become available, if applicable, yet this service does not include their disclosure of the diagnosis, nor the referral for metabolic testing, and interventions. Considering the importance of care being perceived and experienced as continuous and coherent to the patient, we could not justify introducing this additional informational burden to the family
I understand what concerns brought me to the clinical genetics service	I understand what concerns brought my family to do genetics testing	
I understand the reasons why my doctor referred me to the clinical genetics service	I understand the reasons why my doctor may have to refer my family to the clinical genetics service	
I can explain what the condition means to people in my family who may need to know	I can explain what the neurodevelopmental condition means to people in my family who may need to know	Individuals with ASD or DD/ID may have other co‐morbidities and are undergoing investigations for other health conditions. Specifying neurodevelopmental condition was needed to ensure that the impact of genetic results was contextualized on the understanding of their child’s neurodevelopmental condition specifically

Abbreviations: ASD, Autism spectrum disorder; CMA, chromosomal microarray; DD/ID, Developmental delay/Intellectual disability.

*Parent stress* was measured using the 10‐item version of the Perceived Stress Scale (PSS‐10 (S. Cohen & Williamson, 1988)). It measures the extent to which situations in the past month are perceived as stressful. The PSS‐10 has been previously shown to have high internal reliability in a sample of the general population (coefficient alpha = 0.85), adequate test–retest reliability, and correlated with life‐event scores and is a better predictor of health and health‐related outcomes than life‐event scores. Within the measure, higher scores indicate higher levels of parental stress.

*Parent distress* was measured using the Distress Thermometer (DT (Haverman et al., 2013)). The DT was developed for parents of a chronically ill child to identify parents most in need of support in their emotional functioning. The DT consists of a visual ‘thermometer’ ranging from 0 (no distress) to 10 (extreme distress) where individuals were instructed to select the number that best described their overall distress. The thermometer score was shown to correlate with anxiety and depression as measured on the Hospital Anxiety and Depression Scale and showed diagnostic utility; it correctly detected 86% of clinical cases of anxiety and depression among parents and ruled out 67% of non‐clinical cases.

Sociodemographic characteristics of interest were caregiver age on study visit, education, and annual household income. These variables were assessed by a caregiver interview using the Family Background Information Questionnaire (FBIQ) (Statistics Canada, Human Resources and Skills Development Canada, 2010).

To examine characteristics of the care pathway in which genetic testing is embedded, information was collected from two sources: (a) a standardized in‐house referral form completed by the referring clinician during a clinical appointment to discuss the child’s condition, which includes a disclosure of the child’s diagnosis and a discussion for the need for a clinical CMA and (b) chart review on standardized forms developed in‐house. Specific information collected on participating families included child’s age on study referral, child’s gender, child diagnosis, and the diagnostic and genetics reports.

### 2.4 Data analysis

Demographic data were examined using descriptive statistics. One‐sample *t* tests were conducted to compare the main outcome measures (mGCOS‐24, DT, and PSS‐10) with published norms.

Internal consistency of the mGCOS‐24 was assessed using Cronbach's α (Gliem & Gliem, 2003). A two‐way random effects model intraclass correlation was calculated as a test–retest reliability assessment of the mGCOS‐24 and was conducted on a sample of 46 participants, who were invited to complete the questionnaire at a second time‐point on an average of 26 weeks following the study visit (Vaz, Falkmer, Passmore, Parsons, & Andreou, 2013). To ensure that the test–retest reliability is valid, we only included participants who did not receive any post‐test genetic counseling within this time period. To examine the factor structure of the mGCOS‐24 against the original GCOS‐24, we performed a similar exploratory factor analysis (EFA) as is reported in the original measure.

Construct validity of empowerment is the degree to which empowerment and another construct that theoretically should be related to empowerment are observed to be related (Terwee et al., 2007). Parent‐reported stress and distress have been widely explored in both neurodevelopmental and genetic conditions generally. Parents of children with these conditions have higher levels of stress and distress as compared to parents of typically developing children (Ashtiani, Makela, Carrion, & Austin, 2014; Baumann, 2010; Cousino & Hazen, 2013; Dinc & Terzioglu, 2006; Gatzoyia et al., 2014; Kuhlthau et al., 2014; Valicenti‐McDermott et al., 2015). Many affected families also report a significant emotional response to the disclosure of genetic results for their child (Ashtiani et al., 2014; Hayeems et al., 2015; Jez, Martin, South, Vanzo, & Rothwell, 2015; Reiff et al., 2015; Wynn et al., 2018). Thus, we hypothesized a significant inverse correlation between a measure of empowerment with that of stress and distress. This would provide evidence for the construct validity of the mGCOS‐24 for use among parents of a child with ASD or DD/ID undergoing first‐tier genetic testing for the condition. To test this hypothesis, we employed Pearson’s r correlations between the scores of the mGCOS‐24 with those of PSS‐10 and with those of DT, respectively.

Pairwise deletion was employed to handle missing data for maximizing all available data. The target sample size calculated as the number of items of the questionnaire multiplied by seven (*n* = 168), which is one recommendation for validating health status questionnaires (Terwee et al., 2007).

## 3 RESULTS

### 3.1 Participants

Out of 241 eligible families referred by clinicians, 113 (46.9%) were enrolled into the G2O Cohort and with the most common reason reported by families who refused research participation was that they were busy and/or overwhelmed by other demands limiting their participation.

To assess the representativeness of the sample—especially in view of the large proportion of families who declined participation—we independently compared key variables between participating and non‐participating families, excluding families who were ineligible for the study. Ethics approval from the REBs allowed for access to minimal data for participants who were informed about the study by the referring clinician. An independent‐samples *t* test showed that child’s age on study referral was not significantly different between those who declined (*M* = 6.02, *SD* = 3.22) versus those who enrolled in the study (*M* = 6.70, *SD* = 3.72), *t* (239) = 1.55, *p* = 0.12. Fisher’s exact tests also showed that there were no significant differences in diagnosis and gender between the families who enrolled in the study versus those who declined participation, *p*s ≥ 0.05 (86% of families who enrolled had a child with ASD versus 76% of families who declined participation; 74% of families who enrolled had a male child versus 76% of families who declined participation).

Characteristics of the *G2O Cohort* (*n* = 113) are presented in Table 2. Most survey respondents were biological mothers to a male child with ASD. The proportion of families reporting household incomes and parent education was lower than the Montreal average (based on the 2016 census, the average total income of households in 2015 was $69,047 and 31% of respondents reported having completed a university degree or higher (Statistics Canada, 2016)) suggesting the clinically integrated protocol succeeded in enrolling a representative sample of families.

**TABLE 2:** Characteristics of the genome to outcome cohort (*n* = 113)

Characteristic	Statistic
Child’s age on study referral in years *M* (*SD*)	6.7 (3.71)
Child’s gender *N* (%)	
Male	84 (74.3)
Female	29 (25.7)
Child’s diagnosis *N* (%)	
ASD	97 (85.8)
DD/ID	16 (14.2)
Number of child’s siblings	
0	31 (27.4)
1	60 (53.1)
2 or more	22 (19.5)
Caregiver’s age on study visit in years *M* (*SD*)	39.3 (7.9)
Caregiver’s relationship to child *N* (%)	
Biological mother	98 (86.7)
Biological father	11 (9.7)
Adoptive mother	4 (3.5)
Marital status *N* (%)	
Married/common law	96 (85.0)
Single/separated/divorced	17 (15.0)
Respondent education background *N* (%)	
High school or College	53 (46.9)
University or post‐secondary	60 (53.1)
Annual household income *N* (%)	
Less than $40,000	34 (30.1)
Between $40,000 and $80,000	35 (30.1)
More than $80,000	43 (38.1)
Missing	1 (0.9)
Referral source N (%)	
Pediatrics	66 (58.4)
Medical genetics	18 (15.9)
Psychology	12 (10.6)
Self‐referred	12 (10.6)
Neurology	5 (4.4)
CMA results available prior to referral	
Yes	36 (31.9)
No	70 (61.9)
Missing	7 (6.2)
Time between study visit and referral in weeks, median (range)	6.4 (1.0‐68.7)
Time between study referral and final diagnostic report in weeks, median (range)	3.3 (−1‐548)

Abbreviations: ASD, Autism spectrum disorder; CMA, chromosomal microarray imaging; DD/ID, Developmental delay/Intellectual disability; *M*, Mean; *SD*, Standard deviation.

We identified one potential bias in the cohort which is the likelihood of over‐representation of families with ASD, relative to DD/ID. It is surprising that this occurred considering that ASD cases typically make up of 60% of referred samples in diagnostic clinics in our context. Because the proportion of those with ASD was not statistically different between families who enrolled versus those who declined participation, a possible explanation to this is that the referring clinicians in this study are more likely to introduce the study to families with a child with ASD compared to other conditions.

Care pathways linked with genetic testing were diverse as expected. At the time of enrollment in the study, 97 participants had a primary diagnosis of ASD and 16 were diagnosed with DD/ID. The referral to the research study was done mostly by pediatricians during a clinical follow‐up in which discussion for CMA was done as part of the disclosure of the child’s clinical diagnosis or a general follow‐up related to the child’s condition. The final clinical diagnostic report was available for children whose diagnostic evaluation for ASD or DD/ID was completed at the two study sites (*n* = 71), which were not necessarily completed by the referring clinician who disclosed the child’s diagnosis. This is consistent with the diagnostic care pathway for ASD in which the clinical diagnosis involves multiple professionals across multiple clinical visits. Where available, the median time between study referral and the final diagnostic report was 3 weeks; the referring clinician saw 75% of families clinically evaluated at the study sites within 12 weeks of the diagnostic report, indicating that the sample consisted of families whose child was recently diagnosed. The genetics report detailing the CMA results was available prior to the referral for the study for 32% of families indicating that they completed the CMA testing prior to study referral; while seven families chose to participate in this study without undergoing CMA testing. Despite the availability of the genetics results for these families at the time of referral to this study, no participant in the study was informed of their genetic results at that time.

**FIGURE 1:**
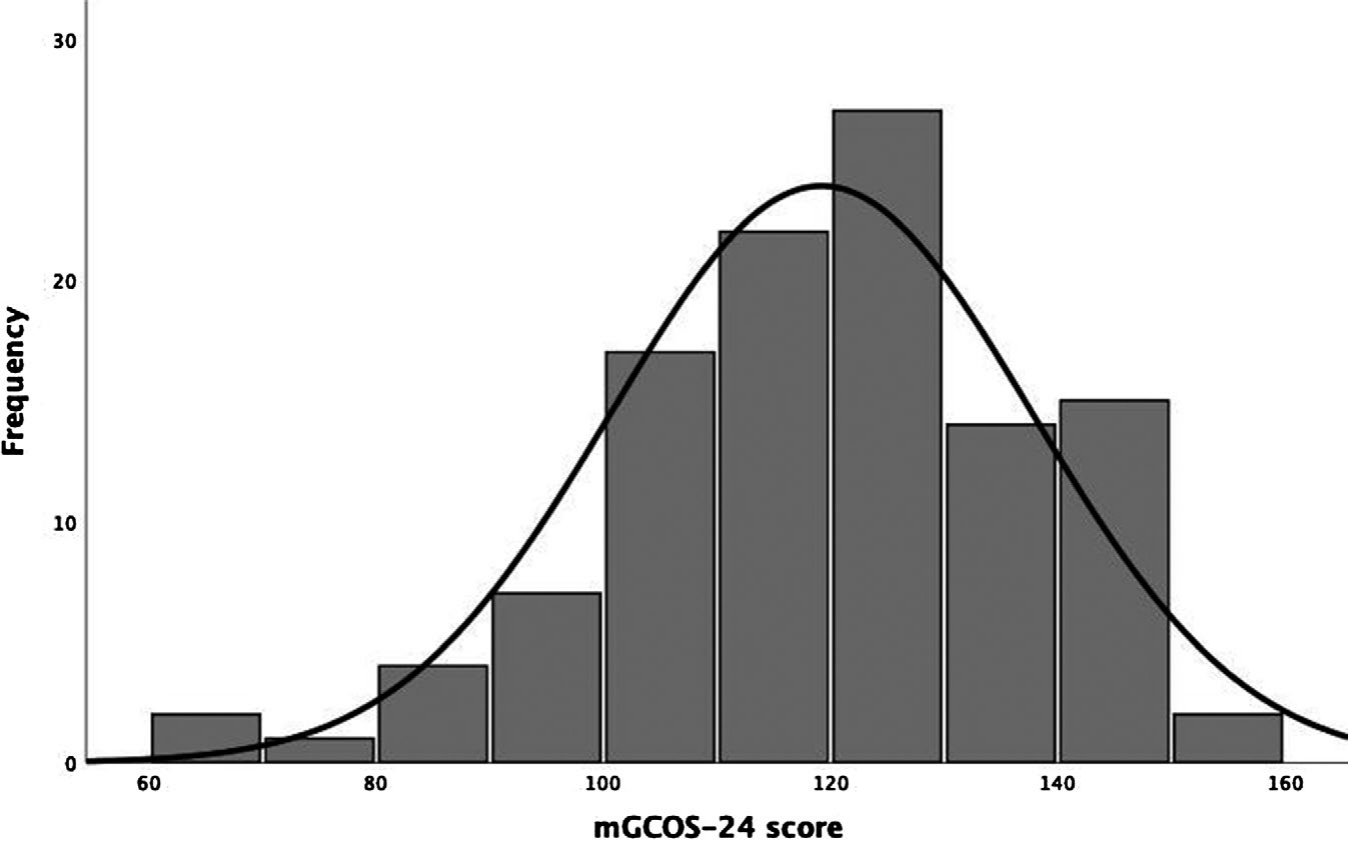
Histogram of mGCOS‐24 total score

Out of the 113 enrolled families, there were two families missing data on both the mGCOS‐24 and PSS‐10 (*n* = 111), and one additional family missing data for the DT (*n* = 110); two families opted out of all questionnaires, and one skipped only the DT. Descriptive statistics of the mGCOS‐24, DT, and PSS‐10 are presented in Table 3. After correcting for multiple comparisons using Bonferroni correction, we found that our sample reported significantly higher total scores on the mGCOS‐24 compared with published scores (*p*s ≤ .002), while the DT and PSS‐10 total scores were not significantly different from norms (*p*s > .0125). This suggests that parents of a child with ASD were reporting greater empowerment following a referral for genetic testing around the time of diagnosis compared with individuals seen at a clinical genetics service. The distribution of the responses on the mGCOS‐24 is presented in Figure 1 with the histogram suggesting a negatively skewed distribution.

**TABLE 3:** Descriptive statistics of main measures for the current study

Measure	N	Min‐Max	Mean (SD)	Norms	One‐sample t test compared to norm
mGCOS‐24 total score	111	61–156	119.2 (18.5)	103.01 (prior to pre‐test counseling) (Thomas & McAllister, 2019)	*t*(110) = 9.20, *p *< .001*
				113.68 (following pre‐test counseling) (Thomas & McAllister, 2019)	*t*(110) = 3.14, *p = *.002*
Distress thermometer	110	0–9	4.0 (2.7)	3.4 (van Oers, Schepers, Grootenhuis, & Haverman, 2017)	*t*(109) = 2.47, *p *= .015
PSS‐10 total score	111	3‐32	17.1 (6.7)	16.14 (Cohen & Janicki‐Deverts, 2012)	*t*(110) = 1.57, *p* = .12

* *p* < .0125.

Participants of the cognitive interview (*n* = 5) were all mothers of boys diagnosed with ASD. The median age of the mothers was 35.1 years (range = 28‐45). The median age of their child with ASD was 5.8 years old (range = 5‐11). Most mothers had a Bachelor’s degree or higher, and all mothers reported an annual household income of more than $80,000. The cognitive interviews of the mGCOS‐24 confirmed that the questionnaire has adequate readability, with parents reporting consistent understanding of the items of the questionnaire.

### 3.2 Factor structure

To determine the factor structure of the mGCOS‐24, we replicated the factor analysis conducted on the original GCOS‐24 (McAllister, Wood, et al., 2011). First, we conducted a maximum likelihood exploratory factor analysis (EFA) with oblique (promax) rotation, using SPSS for Mac version 26 (IBM Corp, 2017). Similar to the original publication (McAllister, Wood, et al., 2011), the data were suitable for EFA: Bartlett’ test was acceptable, χ^2^ = 1074.50, *p* < .001 and the Kaiser‐Meyer‐Olkin test was high (0.81). Results of the EFA are detailed in Table 4.

**TABLE 4:** Results of exploratory factor analysis, showing item communalities and all factor loadings >0.30

	Item	Communalities	1	2	3	4	5	6
9	I am able to cope with having this condition in my family.	0.806	0.816		−0.336			
8	I feel positive about the future.	0.816	0.752	−0.422				
7	I can control how this condition affects my family.	0.52	0.626					
16	I can explain what the condition means to people outside my family who may need to know (e.g., teachers, social workers).	0.718	0.618	0.533				
2	I can explain what the neurodevelopmental condition means to people in my family who may need to know.	0.818	0.61	0.641				
15	I know how to get the non‐medical help I/ my family needs (e.g., educational, financial, social support).	0.497	0.605	0.309				
20	I am able to make plans for the future.	0.65	0.601		−0.374			
3	I understand the impact of the condition on my child(ren)/any child I may have.	0.561	0.579	0.469				
24	I can make decisions about the condition that may change my child(ren)’s future/ the future of any child(ren) I may have.	0.35	0.521					
11	Having this condition in my family makes me feel anxious.	0.809	0.476	−0.493	0.529			
5	I don’t know where to go to get the medical help I/ my family need(s).	0.475	0.461		0.333			
17	I don’t know what I can do to change how this condition affects me/ my children.	0.427	0.442		0.397			
1	I am clear in my own mind why my family is having genetic testing.	0.608	0.439	0.584				
4	When I think about the condition in my family, I get upset.	0.396	0.438	−0.377				
6	I can see that good things have come from having this condition in my family.	0.225	0.406					
22	I am powerless to do anything about this condition in my family.	0.453	0.401			0.339		
19	I am hopeful that my children can look forward to a rewarding family life.	0.392	0.386		−0.411			
21	I feel guilty because I (might have) passed this condition on to my children.	0.27	0.339					
23	I understand what concerns brought my family to do genetics testing.	0.421		0.43				0.435
10	I don’t know what could be gained from each of the options available to me.	0.441			0.401	0.35		
12	I don’t know if this condition could affect my other relatives (brothers, sisters, aunts, uncles, cousins).	0.621			0.369	−0.516	0.34	
18	I don’t know who else in my family might be at risk for this condition.	0.367					0.47	
13	In relation to the condition in my family, nothing I decide will change the future for my children/ any children I might have.	0.223					0.31	
14	I understand the reasons why my doctor may have to refer my family to the clinical genetics service.	0.281						0.372

To determine the factor structure from the EFA, we conducted parallel analysis as was done on the original GCOS (McAllister, Wood, et al., 2011). Using a published SPSS syntax (O’connor, 2000), 1000 data matrices were randomly generated. Each matrix consisted of 111 cases and 24 variables. The 1st eigenvalue that emerged from these randomly generated matrices was 1.20. Since the 1st eigenvalue of the real dataset (5.96) exceeded the 1st eigenvalue generated by the parallel analysis (*i.e.,* from a pure chance dataset), this suggests the modified GCOS‐24 has an optimal one‐factor structure, a result similar to the validation of the original measure (McAllister, Wood, et al., 2011).

### 3.3 Reliability analyses

Cronbach’s alpha of the mGCOS‐24 was 0.84 (*n* = 111), which suggests good internal consistency. Intraclass correlation between mGCOS‐24 scores at baseline versus follow‐up at 26 weeks was 0.88, *p* < .001 (*n* = 46), suggesting strong test–retest reliability.

### 3.4 Construct validity

Pearson’s *r* shows that mGCOS‐24 scores were significantly inversely correlated with PSS‐10 scores (*r* = −0.39, *p* < .001, *n* = 111), and with DT scores (*r* = −0.47, *p* < .001, *n* = 110). This provides evidence for construct validity of the mGCOS‐24.

## 4 DISCUSSION

The primary aim of the current study was to validate the mGCOS‐24 for use in assessing the anticipated impact of genetics results among families who have a child with ASD and related conditions. Following adaptation of the GCOS‐24 into the mGCOS‐24 and the implementation of the mGCOS‐24 within a clinically integrated research protocol, we found that mGCOS‐24 showed acceptable levels of internal consistency, test–retest reliability, and construct validity.

This study supports the feasibility and utility of empowerment as a parent‐reported outcome measure of the provision of CMA within a target population of parents of a child with ASD. Despite many families who were offered participation in research opting out, those who did participate exhibited high rates of completing study measures. Even more encouraging is the broad range of socioeconomic status of families enrolled and completing the study.

Due to extensive qualitative (McAllister, Dunn, et al., 2011; McAllister et al., 2008) and validative (McAllister, Wood, et al., 2011) studies in the development of the original GCOS‐24, the assumption that the GCOS‐24 measures empowerment is well‐founded. Thus, we found that the internal consistency of the mGCOS‐24 used within an ASD population was comparable with the original GCOS‐24 (Cronbach’s α = 0.84 vs. 0.87, respectively) (McAllister, Wood, et al., 2011). The test–retest reliability was also similar to that reported in the original GCOS‐24 (0.88 vs 0.86) despite the longer time interval in this study, providing further evidence of the GCOS‐24’s stability.

Evidence of construct validity was supported by the significant correlation between the mGCOS‐24 scores with that of stress and distress. Stress and distress were chosen in this study because they have been widely explored in parents of a child with a neurodevelopment condition, in general and in genetic services in particular. In contrast, the GCOS‐24 was validated against the health locus of control, perceived personal control, anxiety, depression, satisfaction with life, and authenticity (McAllister, Dunn, et al., 2011). Understanding how these constructs are characterized in the target population of parents of a child with neurodevelopmental conditions can inform the extent to which comparing the mGCOS‐24 with the above measures would support its construct validity above and beyond the correlations found with stress and distress.

The mGCOS‐24 empowerment scores in the current study were statistically higher in our target group of parents relative to those recently reported for individuals undergoing pre‐test counseling (Thomas & McAllister, 2019). While this pattern cannot be directly explained based on the current findings, it does highlight the variability in the care pathways linked with genetics testing for ASD around the time of diagnosis. The time around diagnosis is dynamic in this population and marked with variable post‐diagnostic support; thus, this time can be considered as a significant life event (Crane, Chester, Goddard, Henry, & Hill, 2016). Consistently, we found significant variability in both the clinical services using genetic testing as well as in characteristics of the care pathways reported by clinicians. This is consistent with existing evidence that the provision of genetic testing is implemented inconsistently for this target population; some clinicians prepare the families for the likelihood of the limited utility of null or VUS results through pre‐test counseling (Carter & Scherer, 2013), while others do not inform the families of the availability of genetic testing following diagnosis (Wydeven et al., 2012). Notably, while almost all families consent to genetic testing, routine care is highly variable in terms of pre‐test counseling and return of results and perhaps the referral to medical genetics and genetic services. Families whose children will receive a negative finding will not receive any counseling, a pattern that potentially gives rise to some of the negative impacts of testing reported in previous studies (Giarelli & Reiff, 2015; Hayeems et al., 2016; Reiff et al., 2012; Reiff et al., 2015).

Taken together, our findings support the utility of the adapted GCOS‐24 with the target population of ASD and related conditions. Yet, further work to understand empowerment within this population across time and within a well‐characterized routine care pathway is needed to parse out the vital elements of genetics services that lead to an optimal impact.

### 4.1 Limitations and future directions

A main limitation of this study is that we implemented the mGCOS‐24 in the parents of our cohort, but we did not have a valid methodology to obtain first‐person perspectives of children with ASD themselves. While diagnosed individuals were not the target population of the current study, future research will need to rely on alternative methods to capture perspectives of the diagnosed individuals as well (Tesfaye et al., 2019).

Furthermore, our study questions the generalizability of the results to neurodevelopmental conditions other than ASD. Our sample was over‐representative of ASD, which prevented us from statistically comparing the validity of the mGCOS‐24 between the ASD and DD/ID groups. Thus, future research would need to confirm if the mGCOS‐24 can be used among parents whose child is diagnosed with any other neurodevelopmental condition that warrants genetic testing.

Additionally, we did not measure the mGCOS‐24’s sensitivity to change (*i.e.,* its ability to detect ‘clinically important changes’) which is an important element of validating a health questionnaire (Terwee et al., 2007). Future work is needed to assess the measure’s sensitivity to change while accounting for the tremendous variability we identified in routine care pathways for the target population. Further research can also build on our current study to examine other properties related to validity such as concurrent validity and divergent validity.

Lastly, while the sample was representative, the participation rate into the cohort was low. Comparisons of available characteristics between enrolled and non‐enrolled families showed no differences on basic characteristics. However, it is unknown if other sociodemographic factors have affected the families’ likelihood to enroll in the study. Adoption of measures like the mGCOS‐24 in routine services would further enhance representativeness and provide insight into barriers to research participation encountered by families. Further, measurement of empowerment in routine care pathways for ASD and related conditions would offer opportunities to evaluate how variations in these care pathways impact outcomes.

Along these lines, the original developers of the GCOS‐24 recently shortened the GCOS‐24 into the six‐item Genomics Outcomes Scale (GOS) for use in other contexts outside of traditional *clinical genetic services*, for contexts the authors termed ‘mainstreaming genetic testing’ (Grant, Pampaka, Payne, Clarke, & McAllister, 2019). Among the items removed from the original GCOS‐24, in this new measure were the four items we had to revise in the mGCOS‐24 (Table 1). Unfortunately, we could not compute an equivalent GOS score for this population because further changes to positively frame the questions in the GOS were done. Regardless, this shows how our efforts have converged on the need for an adapted outcome measure of the genetics care pathway now ubiquitous in conditions like ASD and that further alignment between the fields of autism and clinical genetics is needed to advance both clinical practice and research in these fields.

In conclusion, we adapted the GCOS‐24 for use with parents of children with ASD. Future work with this population would support improved understanding and optimization of care pathways so that they are evidence‐based and family‐centered. With the advent of more powerful technologies like whole exome/genome sequencing offering greater amounts of dynamic information, the time is now for understanding and optimizing the care pathway integrating genetics services across conditions to ensure that individuals and families receive the best care they deserve.

## AUTHOR CONTRIBUTIONS

Afiqah Yusuf made substantial contributions to the conception and design of the work and the acquisition, analysis, and interpretation of data for the work; drafted the work and revised it critically for important intellectual content; approved the final version to be published; and agreed to be accountable for all aspects of the work in ensuring that questions related to the accuracy or integrity of any part of the work are appropriately investigated and resolved. Iskra Peltekova, Tal Savion‐Lemieux, Stephen W. Scherer, and Mayada Elsabbagh made substantial contributions to the conception and design of the work, revised it critically for important intellectual content; approved the final version to be published; and agreed to be accountable for all aspects of the work in ensuring that questions related to the accuracy or integrity of any part of the work are appropriately investigated and resolved. Jennifer Frei and Jennifer Howe made substantial contributions to the acquisition of data for the work; revised it critically for important intellectual content; approved the final version to be published; and agreed to be accountable for all aspects of the work in ensuring that questions related to the accuracy or integrity of any part of the work are appropriately investigated and resolved. Ridha Joober made substantial contributions to the acquisition and interpretation of data for the work; revised it critically for important intellectual content; approved the final version to be published; and agreed to be accountable for all aspects of the work in ensuring that questions related to the accuracy or integrity of any part of the work are appropriately investigated and resolved.

## Conflicts of interest

Afiqah Yusuf, Iskra Peltekova, Tal Savion‐Lemieux, Jennifer Frei, Ridha Joober, Jennifer Howe, Stephen W. Scherer, and Mayada Elsabbagh declare that they have no conflict of interest.

## Human studies and informed consent

All procedures followed were in accordance with the ethical standards of the Research Ethics Boards of the McGill University Health Centre and of the Douglas Mental Health University Institute and with the Helsinki Declaration of 1975, as revised in 2000. Informed consent was obtained from all participants for being included in the study. The abovementioned REBs authorized the use of minimal de‐identified aggregate data of referred individuals who declined participation in the study.

## Supporting information


**Appendix S1**

## Data Availability

The data that support the findings of this study are available on request from the corresponding author. The data are not publicly available due to privacy or ethical restrictions.
