# Supplementary material for: Adaptation and validation of the Genetic Counseling Outcome Scale for autism spectrum disorders and related conditions
Source: J Genet Couns. 2020 Sep 6;30(1):305–18. doi: 10.1002/jgc4.1323 (PMC7891368; doi:10.1002/jgc4.1323)
Supplement: Supplementary file 1 — Appendix S1 [file JGC4-30-305-s001.docx]

**The modified Genetic Counselling Outcome Scale (mGCOS-24)**

Using the scale below, circle a number next to each statement to indicate how much you agree with the statement. Please answer all the questions. For questions that are not applicable to you,

please choose option 4 (neither agree nor disagree).

strongly disagree

disagree

slightly disagree

neither agree nor disagree

slightly agree

agree

strongly agree

| 1 | I am clear in my own mind why my family is having genetic testing. | 1 | 2 | 3 | 4 | 5 | 6 | 7 |
| --- | --- | --- | --- | --- | --- | --- | --- | --- |
| 2 | I can explain what the neurodevelopmental condition means to people in my family who may need to know. | 1 | 2 | 3 | 4 | 5 | 6 | 7 |
| 3 | I understand the impact of the condition on my child(ren)/any child I may have. | 1 | 2 | 3 | 4 | 5 | 6 | 7 |
| 4 | When I think about the condition in my family, I get upset. | 1 | 2 | 3 | 4 | 5 | 6 | 7 |
| 5 | I don’t know where to go to get the medical help I / my family need(s). | 1 | 2 | 3 | 4 | 5 | 6 | 7 |
| 6 | I can see that good things have come from having this condition in my family. | 1 | 2 | 3 | 4 | 5 | 6 | 7 |
| 7 | I can control how this condition affects my family. | 1 | 2 | 3 | 4 | 5 | 6 | 7 |
| 8 | I feel positive about the future. | 1 | 2 | 3 | 4 | 5 | 6 | 7 |
| 9 | I am able to cope with having this condition in my family. | 1 | 2 | 3 | 4 | 5 | 6 | 7 |
| 10 | I don’t know what could be gained from each of the options available to me. | 1 | 2 | 3 | 4 | 5 | 6 | 7 |
| 11 | Having this condition in my family makes me feel anxious. | 1 | 2 | 3 | 4 | 5 | 6 | 7 |
| 12 | I don’t know if this condition could affect my other relatives (brothers, sisters, aunts, uncles, cousins). | 1 | 2 | 3 | 4 | 5 | 6 | 7 |
| 13 | In relation to the condition in my family, nothing I decide will change the future for my children / any children I might have. | 1 | 2 | 3 | 4 | 5 | 6 | 7 |
| 14 | I understand the reasons why my doctor may have to refer my family to the clinical genetics service. | 1 | 2 | 3 | 4 | 5 | 6 | 7 |
| 15 | I know how to get the non-medical help I / my family needs (e.g. educational,  financial, social support). | 1 | 2 | 3 | 4 | 5 | 6 | 7 |
| 16 | I can explain what the condition means to people outside my family who may need to know (e.g. teachers, social workers). | 1 | 2 | 3 | 4 | 5 | 6 | 7 |
| 17 | I don’t know what I can do to change how this condition affects me / my children. | 1 | 2 | 3 | 4 | 5 | 6 | 7 |
| 18 | I don’t know who else in my family might be at risk for this condition. | 1 | 2 | 3 | 4 | 5 | 6 | 7 |
| 19 | I am hopeful that my children can look forward to a rewarding family life. | 1 | 2 | 3 | 4 | 5 | 6 | 7 |
| 20 | I am able to make plans for the future. | 1 | 2 | 3 | 4 | 5 | 6 | 7 |
| 21 | I feel guilty because I (might have) passed this condition on to my children. | 1 | 2 | 3 | 4 | 5 | 6 | 7 |
| 22 | I am powerless to do anything about this condition in my family. | 1 | 2 | 3 | 4 | 5 | 6 | 7 |
| 23 | I understand what concerns brought my family to do genetics testing. | 1 | 2 | 3 | 4 | 5 | 6 | 7 |
| 24 | I can make decisions about the condition that may change my child(ren)’s future / the future of any child(ren) I may have. | 1 | 2 | 3 | 4 | 5 | 6 | 7 |
